# Neglect of Vaccination

**Published:** 1833

**Authors:** 


### VI. Neglect of Vaccination—Free Schools.

The neglect of *Vaccination*, in Ohio, especially by the poorer classes of people, is, or ought to be, most alarming. Might not our legislature terminate this criminal apathy? We think they might; and hope they will, for at no distant day, we are likely to have the Natural Small Pox superadded to the Epidemic Cholera. Our plan is, that a law should be passed, excluding every child from the benefits of our admirable system of free schools, who has not been Vaccinated. To prevent complaints, it should not take effect, till six months after its passage, which would afford ample time to have all the children of the state Vaccinated; and, that those who are *very* poor, for whom the schools were, indeed, chiefly designed, might not be excluded, it should be made the duty of the Overseers of the poor, on the application of parents who have not the means of paying for the operation, to have it done at the public expense.

The immediate impulse and permanent stimulus, which this regulation would impart to the practice of Vaccination, would, we doubt not, render it universal over the state; and we respectfully commend it to the consideration of newspaper editors, politicians, (meaning statesmen) and the benevolent of all sects and parties.

